# Analysis of intracellular tyrosine phosphorylation in circulating neutrophils as a rapid assay for the *in vivo* effect of oral tyrosine kinase inhibitors

**DOI:** 10.3389/fphar.2023.1056154

**Published:** 2023-04-06

**Authors:** Krisztina Futosi, Boglárka Bajza, Dorottya Deli, András Erdélyi, Simon Tusnády, Attila Mócsai

**Affiliations:** ^1^ Department of Physiology, Semmelweis University School of Medicine, Budapest, Hungary; ^2^ ELKH-SE Inflammation Physiology Research Group, Eötvös Loránd Research Network, Budapest, Hungary

**Keywords:** neutrophils, tyrosine kinases, intracellular tyrosine phosphorylation, tyrosine kinase inhibitors (TKIs), rapid *in vivo* assay

## Abstract

Tyrosine kinases are crucial signaling components of diverse biological processes and are major therapeutic targets in various malignancies and immune-mediated disorders. A critical step of development of novel tyrosine kinase inhibitors is the transition from the confirmation of the *in vitro* effects of drug candidates to the analysis of their *in vivo* efficacy. To facilitate this transition, we have developed a rapid *in vivo* assay for the analysis of the effect of oral tyrosine kinase inhibitors on basal tyrosine phosphorylation of circulating mouse neutrophils. The assay uses a single drop of peripheral blood without sacrificing the mice. Flow cytometry using intracellular staining by fluorescently labeled anti-phosphotyrosine antibodies revealed robust basal tyrosine phosphorylation in resting circulating neutrophils. This signal was abrogated by the use of isotype control antibodies or by pre-saturation of the anti-phosphotyrosine antibodies with soluble phosphotyrosine amino acids or tyrosine-phosphorylated peptides. Basal tyrosine phosphorylation was dramatically reduced in neutrophils of triple knockout mice lacking the Src-family tyrosine kinases Hck, Fgr, and Lyn. Neutrophil tyrosine phosphorylation was also abrogated by oral administration of the Abl/Src-family inhibitor dasatinib, a clinically used anti-leukemic agent. Detailed dose-response and kinetic studies revealed half-maximal reduction of neutrophil tyrosine phosphorylation by 2.9 mg/kg dasatinib, with maximal reduction observed 2 h after inhibitor administration. Taken together, our assay allows highly efficient analysis of the *in vivo* effect of orally administered tyrosine kinase inhibitors, and may be used as a suitable alternative to other existing approaches.

## Introduction

Tyrosine kinases phosphorylate tyrosine residues on intracellular proteins, leading to conformational changes, altered enzymatic activity and new protein-protein interaction capabilities of their target proteins (Hubbard and Till, 2000; Fabbro and McCormick, 2006; Blaukat et al., 2007). Mammals express approx. 90 tyrosine kinases which can be divided into receptor and non-receptor (cytoplasmic) tyrosine kinases (Hubbard and Till, 2000; Lemmon and Schlessinger, 2010; Schlessinger, 2014; Espada and Martin-Perez, 2017). Tyrosine kinases are involved in nearly all biological processes including various aspects of signal transduction such as regulation of cell cycle and proliferation, cellular adhesion, growth factor signaling or immunoreceptor signal transduction. Given their role in cellular proliferation and immune cell function, they play central roles in various pathological processes such as tumor development and progression, or autoimmune and inflammatory diseases (Paul and Mukhopadhyay, 2004; Fabbro and McCormick, 2006; Mulligan, 2014; Schwartz et al., 2017; Lipsick and History of Cancer Research, 2019; Szilveszter et al., 2019; Dawson et al., 2021; Tanaka et al., 2022). Accordingly, tyrosine kinases have become major therapeutic targets in malignant and immune-mediated diseases (Paul and Mukhopadhyay, 2004; Fabbro and McCormick, 2006; Mulligan, 2014; Schwartz et al., 2017; Lipsick and History of Cancer Research, 2019; Szilveszter et al., 2019; Cohen et al., 2021; Dawson et al., 2021; Tanaka et al., 2022). There is also a major interest from the pharmaceutical industry to develop novel tyrosine kinase inhibitors for various human diseases.

Preclinical development of tyrosine kinase inhibitors for therapeutic purposes proceeds through of a number of consecutive steps (Chung et al., 2004; Saeidnia et al., 2015; Brake et al., 2017). The first steps include conceptualization (possibly involving *in silico* screening), chemical synthesis and analysis in various *in vitro* cellular assays. Most promising candidates then progress to further optimization and analysis *in vivo* animal models (Chung et al., 2004; Saeidnia et al., 2015; Brake et al., 2017).

The transition from *in vitro* to *in vivo* analyses is a critical step during drug development (Chung et al., 2004; Hughes et al., 2011; Saeidnia et al., 2015; Brake et al., 2017). The pharmacokinetic properties of drug candidates may be difficult to predict and may require extensive *in vitro* and *in vivo* testing. Drug candidates able to exert promising effects *in vitro* cellular assays may have less favorable *in vivo* characteristics such as limited oral availability, unexpected interaction with serum components or rapid elimination through metabolism or excretion (Sun et al., 2022). All these need to be tested, either through complex and often poorly predictable *in vitro* ADME-Tox approximation assays or by expensive and time-consuming *in vivo* pharmacokinetic and pharmacodynamic (functional) studies (Glassman and Muzykantov, 2019). *In vivo* studies are hindered by the need for larger drug quantities, as well as the difficulties of the analysis of drug concentrations in complex biological fluids (Peers et al., 2014; Dahlin et al., 2015). In addition, the drug concentrations alone may not entirely reflect the *in vivo* effects of the inhibitors. These difficulties provide substantial obstacles for the transition of *in vitro* to *in vivo* analysis of tyrosine kinase inhibitors.

A number of tyrosine kinase inhibitors target cells of the hematopoietic or immune compartments (Moen et al., 2007; Quintas-Cardama et al., 2009; Lindauer and Hochhaus, 2010; Iqbal and Iqbal, 2014; Dhillon, 2017; Keating, 2017; Yamaoka, 2019). They include inhibitors against hematological malignancies such as chronic myeloid leukemia and other leukemias/lymphomas (Moen et al., 2007; Quintas-Cardama et al., 2009; Lindauer and Hochhaus, 2010; Iqbal and Iqbal, 2014; Keating, 2017), or inhibitors targeting the immune/inflammatory response, e.g., during autoimmune or inflammatory diseases (Dhillon, 2017; Yamaoka, 2019). It is reasonable to assume that such inhibitors reduce tyrosine phosphorylation in circulating leukocytes such as neutrophils.

We have previously reported that isolated mouse bone marrow (Mócsai et al., 2002) or human peripheral blood (Futosi et al., 2012) neutrophils show substantial basal tyrosine phosphorylation of a number of proteins by an immunoblotting approach, even under apparently resting conditions. Our limited prior experiments also revealed reduced basal tyrosine phosphorylation of neutrophils from triple knockout mice lacking three Src-family kinases (Hck, Fgr, and Lyn) expressed in the myeloid compartment (Mócsai et al., 2002), or upon the *in vitro* administration of the Abl/Src-family tyrosine kinase inhibitor dasatinib (Futosi et al., 2012). However, this approach has several limiting factors, such as the tedious neutrophil isolation and immunoblotting procedures, difficulties in signal quantification, as well as sacrificing the mice. There are available other methods which are suitable for identifying phosphorylation sites from individual proteins/protein-complexes or global phosphorylation sites from whole cell lysates. One of the most widespread approach to detect phosphorylation sites is the mass spectrometry-based proteomics of the intracellular proteins (e.g., LC-MS/MS), while as an alternative way, peptide-based micro-, and macroarrays are widely employed for the analysis kinase activity (Breitkopf and Asara, 2012; Kozlov et al., 2012; Deng and Turk, 2016; Lai et al., 2016; Zhang et al., 2017). In spite of the high sensitivity and specificity of the latter mentioned methods, the whole procedure from cell/tissue isolation and lysis to fractionation, purification and enrichment of phosphoproteins or kinases is long and costly (Breitkopf and Asara, 2012; Kozlov et al., 2012; Deng and Turk, 2016; Lai et al., 2016; Zhang et al., 2017).

Other limitations of those above mentioned approaches included the potential changes of tyrosine phosphorylation during the cell isolation procedure and the potential differences between bone-marrow and circulating neutrophils. Others have reported the analysis of tyrosine phosphorylation of intracellular molecules at the single-cell level using intracellular flow cytometry, as a rapid and quantitative assay from very small sample quantities (Far et al., 1994; Vuillier et al., 1995; Muller et al., 1996; Hubert et al., 1997; Yoon et al., 1997; Muller et al., 1998; Desplat et al., 2004; Perez and Nolan, 2006; Guerrouahen et al., 2009; Matamoros-Volante et al., 2018; Hao et al., 2022; Prendecki et al., 2023).

The difficulties of transition from *in vitro* to *in vivo* assays during tyrosine kinase inhibitor drug development, along with the reported basal tyrosine phosphorylation of neutrophils and the possibility for intracellular flow cytometry, prompted us to develop a novel assay for the rapid quantitative analysis of the effect of tyrosine kinase inhibitors in experimental mice. Our assay detects basal intracellular tyrosine phosphorylation in neutrophils from a single drop of blood using intracellular flow cytometry without a resource- and time-consuming isolation procedure. We provide a detailed description of this assay, along with validation using antibody competition assays, genetically modified mouse strains and detailed analysis of the effect of the clinically used tyrosine kinase inhibitor dasatinib. Our results suggest that flow cytometric analysis of neutrophil tyrosine phosphorylation may be used as a suitable alternative approach for the rapid analysis of the *in vivo* effects of novel tyrosine kinase inhibitors.

## Materials and methods

### Mice

Wild-type C57BL/6 mice were originally purchased from the Jackson Laboratory and then bred in our animal care facility. Triple knockout mice homozygous for the *Hck* ^tm1Hev^, *Fgr* ^*tm1Hev*^, and *Lyn* ^tm1Sor^ mutations (referred to as *Hck* ^−/−^ *Fgr* ^−/−^ *Lyn* ^−/−^ mice) on the C57BL/6 genetic background were described previously (Kovács et al., 2014). The *Hck* ^−/−^ *Fgr* ^−/−^ *Lyn* ^−/−^ mice were bred in homozygous form and their genotype was sporadically confirmed by allele-specific PCR.

All mice were bred and maintained in individually sterile ventilated cages (Tecniplast) in a specific pathogen-free facility and transferred to a conventional facility for experiments. Mice of both genders were used at 2–6 months of age. Animals were fed with normal commercial diet (Ssniff S8189-S095). All animal experiments were done at Semmelweis University Department of Physiology and approved by the Animal Experimentation Review Board of Semmelweis University (approval number: PE/EA/00284–7/2021).

### Dasatinib treatment

Where indicated, the mice were treated with 1, 2, 5, 10, 20 or 50 mg/kg dasatinib (Selleckchem) suspended in a 1:2 mucilago:water mixture during the morning by oral gavage. Control mice received vehicle as control. The time of dasatinib or vehicle administration was defined as the 0 time point. Blood samples were collected from the experimental animals immediately before (0 h) as well as 2, 5, 8 and/or 24 h after dasatinib/vehicle administration. All mice were tested once and were not re-used beyond taking multiple blood samples from the same mice within the 24-h timeframe of the experiments in Figure 7.

### Sample preparation and staining

Peripheral blood samples were collected from each mouse during the morning or at the indicated time points after dasatinib/vehicle administration. A drop of blood was taken from each mouse by tail incision, and placed immediately in ice-cold PBS supplemented with 5% FBS and 50 IU/mL heparin.

Heparinized blood samples were stained with 0.2 μg/mL PerCP-Cy5.5-conjugated anti-Ly6G antibodies (clone 1A8; BD Pharmingen). After 45 min incubation on ice in the dark, the cells were washed and then fixed and permeabilized using the eBioscience Intracellular Fixation and Permeabilization Buffer kit according to the manufacturer’s instructions, also allowing lysis of red blood cells. After washing, the permeabilized cells were stained with FITC-conjugated PY20 anti-phosphotyrosine antibodies (Southern Biotech), FITC-conjugated 4G10 anti-phosphotyrosine antibodies (Millipore) or FITC-conjugated isotype control (clone GC198; Millipore) antibodies, all at a final concentration of 2.5 μg/mL. After incubation for 45 min on ice in the dark, the cells were washed and re-suspended in FACS lysing solution (BD Biosciences).

The specificity of the staining was also confirmed in a competitive assay by pretreatment of the anti-phosphotyrosine antibodies with 4 mM or the indicated concentrations of soluble O-phospho-L-tyrosine (referred to as soluble pTyr) or 4 mM tyrosine-phosphorylated phospho-peptide (RRLIEDAEpYAARG, referred to as pTyr peptide; both from Sigma) for 30 min on ice. Staining was then performed using these antigen presaturated antibodies.

### Flow cytometry

Fluorescence analysis was performed on a BD Biosciences FACSCalibur flow cytometer. Data files were processed by the BD CellQuest and *DeNovo* FCS Express softwares. To eliminate events from non-leukocyte debris (e.g., RBC remnants) the FSc threshold was increased during flow cytometry without interfering with the putative neutrophil gate. Ly6G-positive cells with forward and side scatter characteristics typical of neutrophils were identified as neutrophils. Fluorescence values reflecting intracellular phosphotyrosine levels within those cells were then determined in the respective channel and expressed as the geometric mean fluorescence intensity (MFI) for the given neutrophil population. Isotype control-stained samples were used to obtain non-specific reference staining intensities. The number of neutrophils was between 500 and 1,500 in the majority of the analyzed samples which represented 18%–30% of all leukocytes.

### Presentation of the data and statistical analysis

The number of experiments and the number of the individual mice are indicated in the figure legends for each panel. Unless otherwise stated, quantitative graphs and kinetic curves show mean and SEM from all independent *in vitro* experiments or from all individual mice from the indicated number of *in vivo* experiments. Statistical analyses were carried out using one-way ANOVA (Figure 2) or two-way (factorial) ANOVA, with staining and pTyr/pTyr peptide treatment (Figure 3) or staining and genotype/inhibitor treatment (Figures 4–7) being the two independent variables. On Figure 6B, the difference between 5, 10, 20 and 50 mg/kg dasatinib treatment compared to vehicle treatment were further analyzed by using Dunnett’s *post hoc* test. Unless otherwise stated, *p* values mentioned in the text refer to the difference between anti-pTyr-stained wild-type vs. *Hck* ^−/−^ *Fgr* ^−/−^ *Lyn* ^−/−^, or vehicle vs. dasatinib-treated samples. *p* values below 0.05 were considered statistically significant.

For generation of the percent response values, the responses were expressed in percent of the values of the anti-phosphotyrosine-stained wild-type or vehicle-treated samples. Quantitative graphs show mean and SEM from all individual mice from the indicated number of experiments. Percent inhibition values were calculated by subtracting fluorescence values of isotype control-treated samples and then expressed as percentage of wild-type or vehicle-treated samples. IC_50_ and ED_50_ values were calculated using an online toolkit (www.aatbio.com).

## Results

### Overview of the proposed assay principles

**FIGURE 1:**
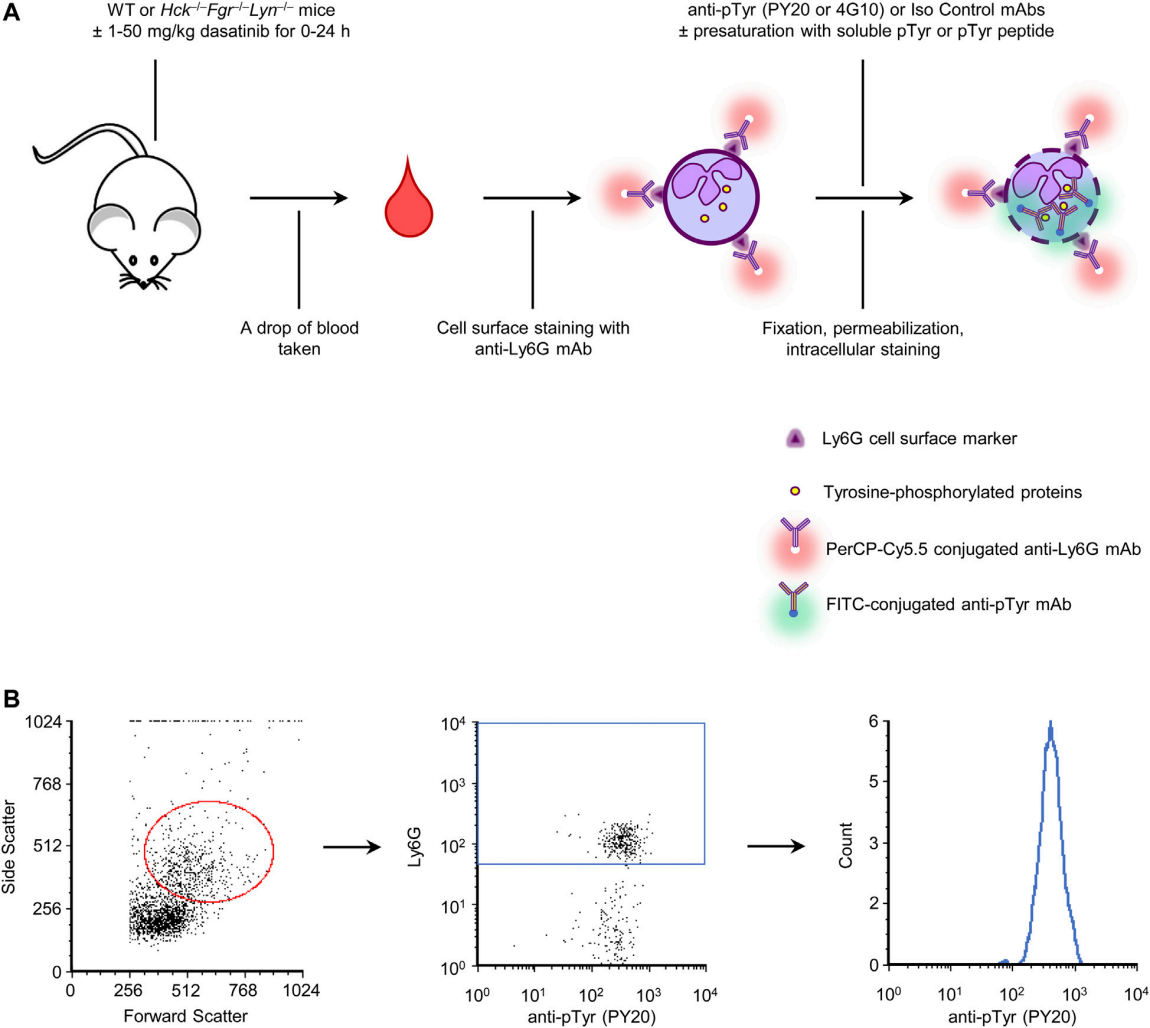
Assay overview and gating strategy. A drop of blood was stained for a neutrophil marker and for intracellular tyrosine phosphorylated proteins. A number of control and validation experiments were also performed, as indicated **(A)**. Neutrophils were identified based on their typical forward and side scatted characteristics and positive staining for the Ly6G neutrophil marker. Tyrosine phosphorylation within those cells was then determined by intracellular flow cytometry using fluorescently labeled anti-phosphotyrosine antibodies **(B)**. WT, wild-type; pTyr, phosphotyrosine; mAb, monoclonal antibody.

We have set up a rapid single-cell-based quantitative assay to test tyrosine kinase inhibitors in experimental mice by intracellular flow cytometry using anti-phosphotyrosine antibodies. To this end, a drop of blood obtained from untreated wild-type mice was stained with fluorescently labeled Ly6G antibodies to stain neutrophils, followed by fixation, permeabilization and intracellular staining using anti-phosphotyrosine antibodies labeled with a different fluorochrome. We have tested two separate anti-phosphotyrosine antibodies (clones PY20 and 4G10) during the experiments, along with an isotype-matched irrelevant control antibody. The overview of the assay protocol is shown in Figure 1A.

In additional experiments, we have also used anti-phosphotyrosine antibodies presaturated with soluble phosphotyrosine amino acids or short tyrosine-phosphorylated peptides, compared wild-type with *Hck* ^−/−^ *Fgr* ^−/−^ *Lyn* ^−/−^ mice, and tested the effect of oral administration of the tyrosine kinase inhibitor dasatinib. Overview of those interventions are also shown in Figure 1A.

Cell-surface staining of peripheral blood samples with anti-Ly6G antibodies and intracellular staining with anti-phosphotyrosine antibodies was followed by flow cytometric analysis. Neutrophils were identified based on their forward- and side-scatter characteristics and positive staining for the Ly6G marker. Tyrosine phosphorylation was then determined within this population. The gating strategy is shown in Figure 1B.

### Flow cytometric analysis of basal tyrosine phosphorylation of neutrophils

**FIGURE 2:**
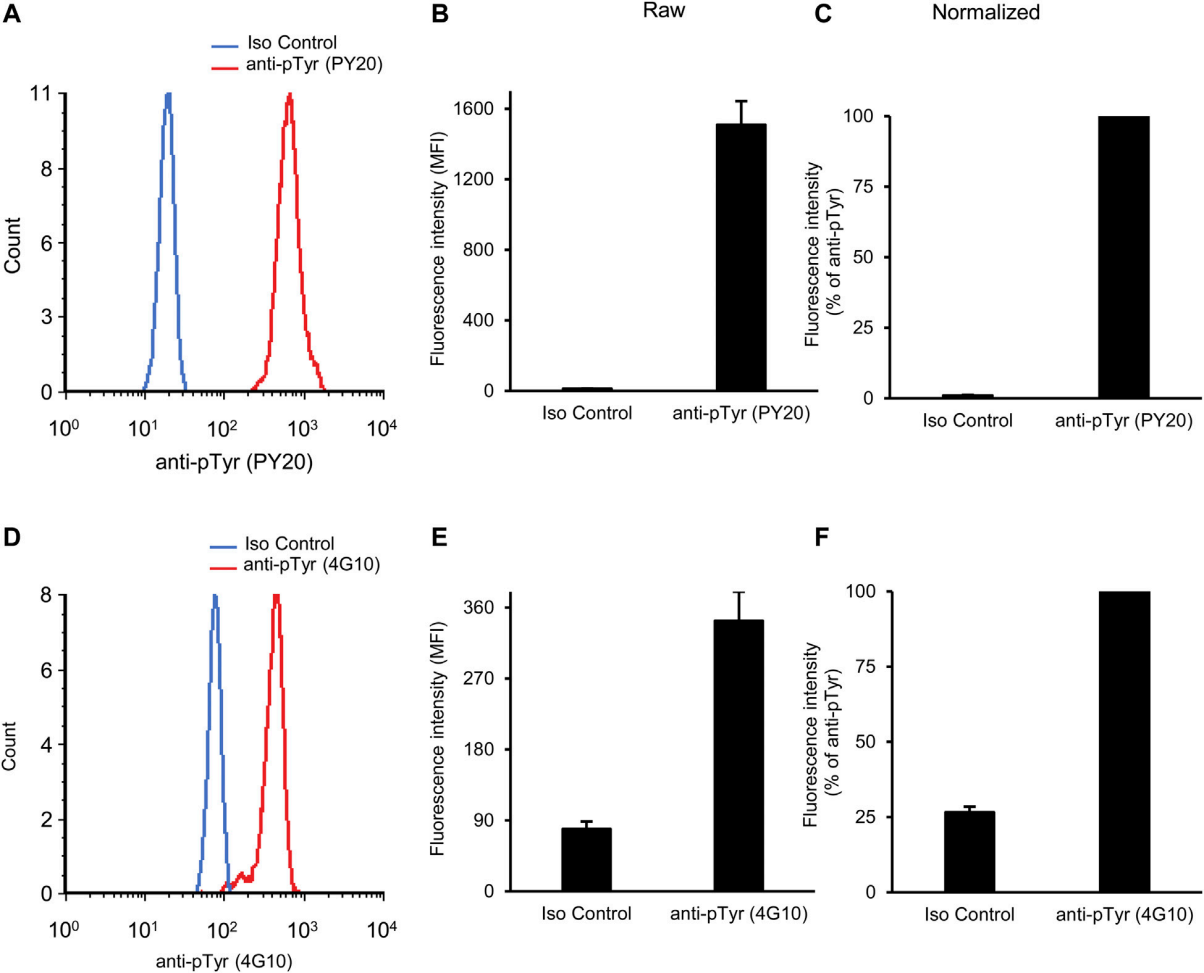
Basal tyrosine phosphorylation of circulating neutrophils. Blood was collected from untreated wild-type mice and stained with the PY20 **(A–C)** or the 4G10 **(D–F)** anti-phosphotyrosine antibodies along with isotype controls. Representative flow cytometric histograms **(A, D)**, raw quantitative fluorescence values expressed as geometric mean fluorescence intensity **(B, E)** and fluorescence values normalized to those of anti-phosphotyrosine-stained samples **(C, F)** are shown. Bar graphs show mean and SEM from 14 **(B–C)** or 71 **(E–F)** mice from 8 **(B–C)** or 28 **(E–F)** independent experiments. MFI, geometric mean fluorescence intensity; pTyr, phosphotyrosine; Iso, isotype.

Figure 2 shows initial results of basal tyrosine phosphorylation of neutrophils. As shown in Figure 2A, a robust phosphotyrosine signal could be observed in neutrophils stained with the PY20 anti-phosphotyrosine antibody, whereas a very low fluorescence signal (supposedly auto-fluorescence or non-specific staining) was seen in isotype control antibody-stained samples. Quantitative measurements (Figure 2B) also revealed a robust increase of fluorescence intensity between isotype control- and PY20 antibody-stained samples (*p* = 2.1 × 10^−11^). For a better comparison between a large number of experimental conditions, we have also calculated the normalized fluorescence intensity, expressed in percent of the signal of PY20-treated samples (Figure 2C). This calculation revealed a 98-fold increase of the fluorescence intensity between the isotype control- and PY20-stained samples.

We have also tested another widely used anti-phosphotyrosine antibody (clone 4G10) in our assay system. As shown in Figures 2A, D difference between isotype control and 4G10 antibody-treated samples could also be readily detected here. However, this difference was not nearly as robust as when the PY20 anti-phosphotyrosine antibody was used. This was also reflected in the raw (Figure 2E; *p* = 1.8 × 10^−10^) and normalized (Figure 2F) data of quantitative measurements. The latter quantification revealed a 3.8-fold average difference between the isotype control- and 4G10-stained samples.

Taken together, our assay is apparently able to detect basal tyrosine phosphorylation of circulating mouse neutrophils, with substantially better signal-to-noise ratio with the PY20 than with the 4G10 antibodies.

To simplify the presentation, only fluorescence values normalized to phosphotyrosine antibody-treated samples will be shown in the following sections.

### Competition by soluble phosphotyrosine or a tyrosine-phosphorylated peptide

**FIGURE 3:**
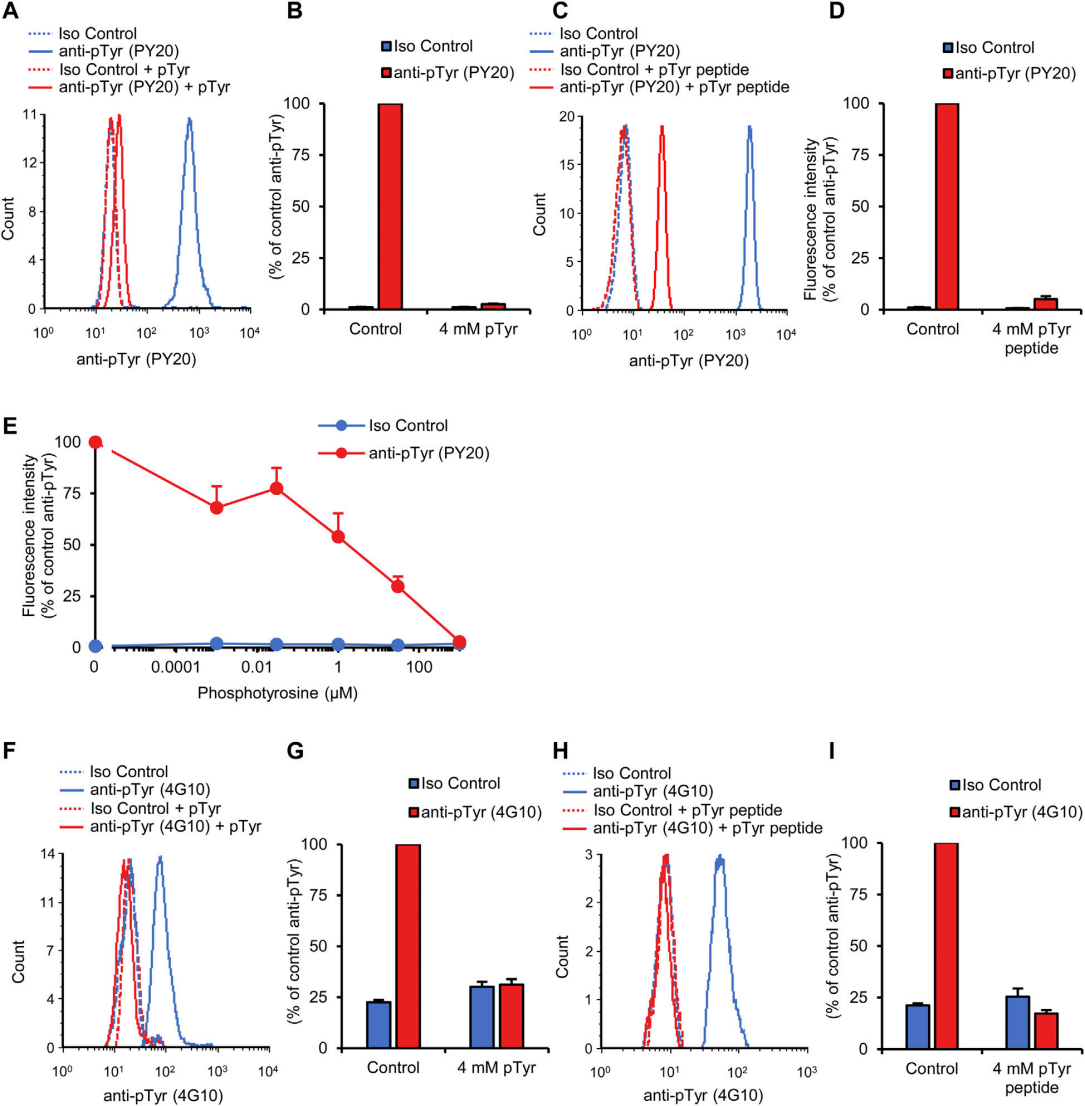
Competition by soluble phosphotyrosine or a tyrosine-phosphorylated peptide. Blood was collected from untreated wild-type mice and stained with the PY20 **(A–E)** or the 4G10 **(F–I)** anti-phosphotyrosine antibodies along with isotype controls. Where indicated, the anti-phosphotyrosine antibodies were presaturated with the indicated concentration of soluble phosphotyrosine **(A, B, and E–G)** or a tyrosine-phosphorylated phosphopeptide **(C, D, H, and I)**. Representative flow cytometric histograms **(A, C, F, and H)** and geometric mean fluorescence intensity values normalized to those of samples stained with anti-phosphotyrosine antibodies without any presaturation with their antigens **(B, D, E, G, and I)** are shown. Quantitative curves and bar graphs show mean and SEM from 17 to 18 **(B)**, 5–6 **(D)**, 6–14 **(E)**, 24 **(G),** and 6 **(I)** mice from 9 **(B)**, 3 **(D)**, 7 **(E)**, 8 **(G)**, and 4 **(I)** independent experiments. pTyr, phosphotyrosine; Iso, isotype.

To confirm that our flow cytometric signal is indeed caused by specific binding of anti-phosphotyrosine antibodies to their cognate antigens, we attempted to saturate the anti-phosphotyrosine antibodies with soluble phosphotyrosine amino acids. As shown in Figure 3A, pretreatment of the PY20 antibodies with 4 mM phosphotyrosine amino acids led to the dramatic reduction of the fluorescence signal which approached that of isotype control-stained samples (*p* = 1.1 × 10^−16^). On the other hand, soluble phosphotyrosine amino acids did not affect the supposedly non-specific signal in isotype control-stained samples. Quantification of the normalized fluorescence intensities (Figure 3B) revealed that 4 mM phosphotyrosine treatment caused a 98.6% reduction in signal strength.

Next we used another competition approach to confirm the specific binding of the anti-phosphotyrosine antibodies to their cognate antigens. In these experiments, the PY20 antibodies have been pretreated with a short (13-amino-acid) tyrosine-phosphorylated phosphopeptide. As shown in Figures 3C, D, pretreatment with 4 mM phosphopeptide also dramatically reduced the fluorescence intensity in our neutrophil tyrosine phosphorylation assay (95.5% inhibition; *p* = 7.0 × 10^−9^).

We have also performed a dose-response analysis of the effect of soluble phosphotyrosine amino acids on the neutrophil tyrosine phosphorylation signal determined using the PY20 antibodies. As shown in Figure 3E, increasing soluble phosphotyrosine amino acid concentrations caused a gradual reduction in fluorescence signal intensity with an IC_50_ of approx. 12 μM (*p* = 1.2 × 10^−15^).

We also tested the effect of soluble antigens on the fluorescence signal obtained using the 4G10 anti-phosphotyrosine antibodies. As shown in the histogram in Figure 3F and quantified in Figure 3G, 4 mM phosphotyrosine amino acids also dramatically reduced the fluorescence signal in this case (*p* = 3.0 × 10^−13^). In addition, tyrosine phosphorylation of neutrophils measured with the 4G10 antibody was also strongly reduced by 4 mM tyrosine-phosphorylated phosphopeptide (Figures 3H, I; *p* = 8.9 × 10^−5^).

The above competition experiments indicate that our flow cytometric approach is indeed specific for tyrosine phosphorylation within circulating neutrophils.

### Reduced phosphorylation in *Hck* ^−/−^ *Fgr* ^−/−^ *Lyn* ^−/−^ neutrophils

**FIGURE 4:**
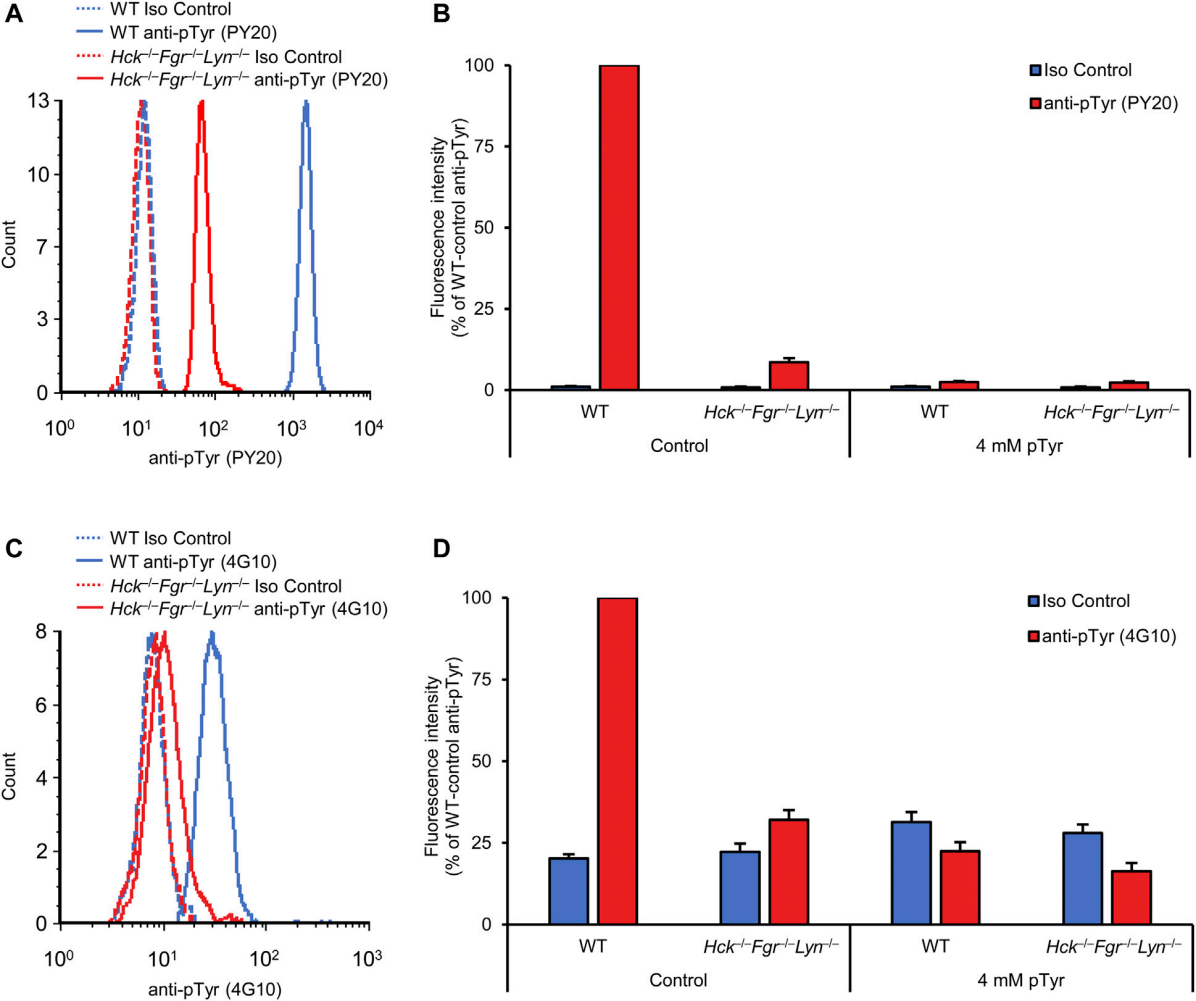
Reduced basal tyrosine phosphorylation in *Hck* ^−/−^ *Fgr* ^−/−^ *Lyn* ^−/−^ neutrophils. Blood samples were obtained from untreated wild-type (WT) or *Hck* ^−/−^ *Fgr* ^−/−^ *Lyn* ^−/−^ mice and stained with the PY20 **(A, B)** or the 4G10 **(C, D)** anti-phosphotyrosine antibodies along with isotype controls. Where indicated, the anti-phosphotyrosine antibodies were presaturated with 4 mM soluble phosphotyrosine. Representative flow cytometric histograms **(A, C)** or geometric mean fluorescence intensity values normalized to those of WT samples stained with anti-phosphotyrosine antibodies without any presaturation with their antigens **(B, D)** are shown. Bar graphs show mean and SEM from 13 to 14 **(B)** and 10–15 **(D)** mice per genotype from 7 **(B)** and 6 **(C)** independent experiments. pTyr, phosphotyrosine; Iso, isotype.

Using an immunoblot approach, we previously observed reduced basal tyrosine phosphorylation of isolated bone marrow neutrophils in triple knockout cells with a combined genetic deficiency of Hck, Fgr, and Lyn, three Src-family kinases present in neutrophils (Mócsai et al., 2002). We have now used our flow cytometric approach to test whether the *Hck* ^−/−^ *Fgr* ^−/−^ *Lyn* ^−/−^ mutation also affected tyrosine phosphorylation of circulating neutrophils that have not gone through a lengthy isolation procedure. As shown in Figure 4A, *Hck* ^−/−^ *Fgr* ^−/−^ *Lyn* ^−/−^ neutrophils showed a strong, although incomplete, reduction of the fluorescence signal intensity detected by staining with the PY20 anti-phosphotyrosine antibodies. Quantification of the signals (Figure 4B) indicated that the *Hck* ^−/−^ *Fgr* ^−/−^ *Lyn* ^−/−^ mutation greatly decreased the intracellular tyrosine phosphorylation of neutrophils (92.7% reduction; *p* = 2.2 × 10^−12^). However, the phosphotyrosine signal in *Hck* ^−/−^ *Fgr* ^−/−^ *Lyn* ^−/−^ neutrophils was clearly distinguishable from the signal in isotype control-treated samples and could be further reduced by soluble phosphotyrosine competition (Figures 4A, B), suggesting the existence of some residual tyrosine phosphorylation in *Hck* ^−/−^ *Fgr* ^−/−^ *Lyn* ^−/−^ cells.

We have also repeated the same experiments with the 4G10 anti-phosphotyrosine antibodies (Figures 4C, D), which again showed a strong inhibition of neutrophil tyrosine phosphorylation by the *Hck* ^−/−^ *Fgr* ^−/−^ *Lyn* ^−/−^ mutation (*p* = 0.033).

Taken together, our results indicate reduced intracellular tyrosine phosphorylation in *Hck* ^−/−^ *Fgr* ^−/−^ *Lyn* ^−/−^ neutrophils and at the same time provide an independent, genetic validation of our flow cytometric assay. They also suggest the existence of residual tyrosine phosphorylation in *Hck* ^−/−^ *Fgr* ^−/−^ *Lyn* ^−/−^ neutrophils.

### Oral dasatinib treatment inhibits neutrophil tyrosine phosphorylation

The long-term aim of our assay development is to allow the rapid analysis of the *in vivo* effect of novel tyrosine kinase inhibitors, thus facilitating the transition of the development of such inhibitors from *in vitro* to *in vivo* stages. To validate this approach, we have tested the effect of dasatinib, a clinically used tyrosine kinase inhibitor, in our assay system. 50 mg/kg dasatinib was administered orally to wild-type mice, followed by the flow cytometric analysis of the intracellular tyrosine phosphorylation of neutrophils from a drop of blood taken 2 h later.

**FIGURE 5:**
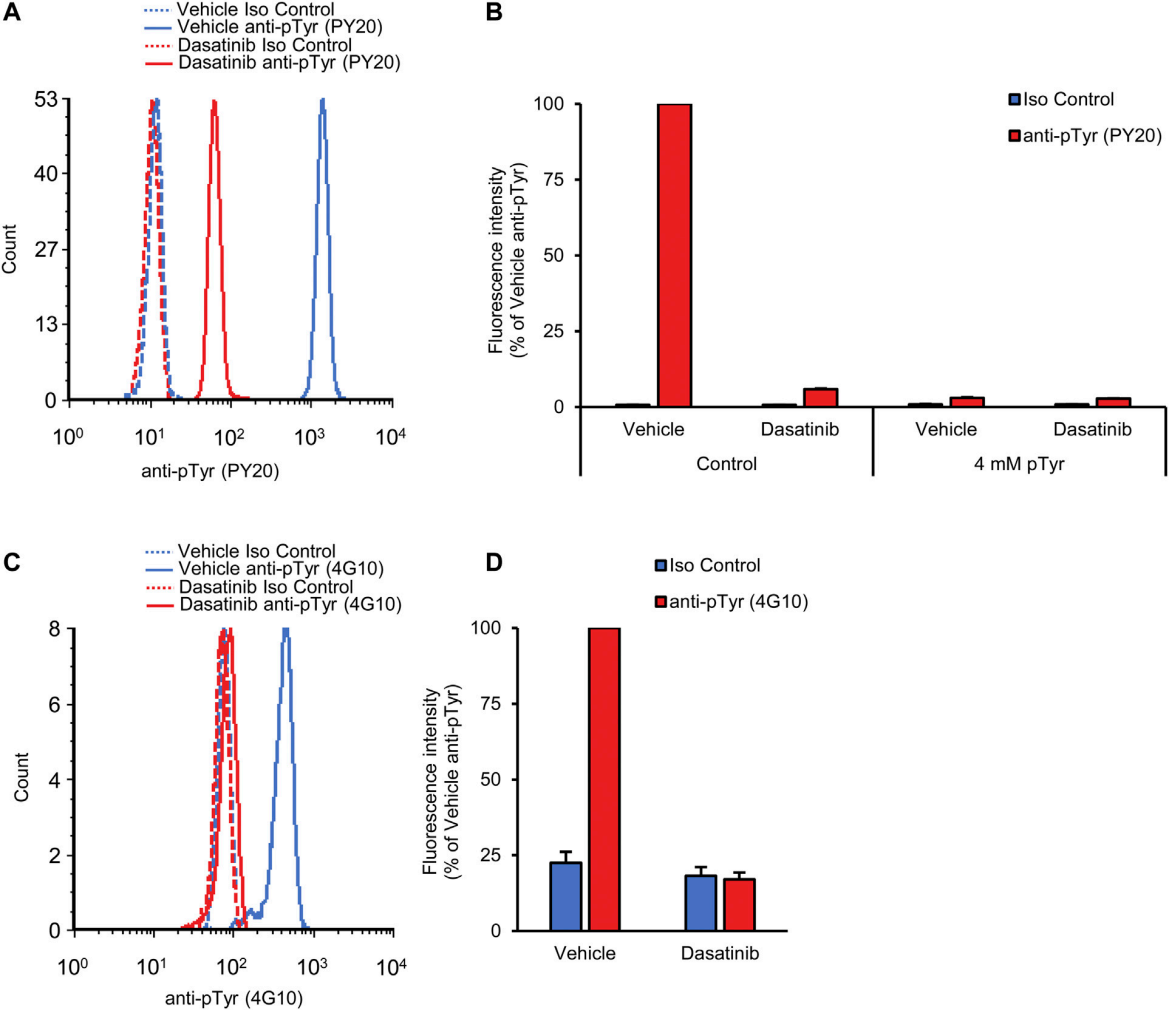
Orally administered dasatinib inhibits neutrophil tyrosine phosphorylation. Wild-type mice were treated with vehicle or 50 mg/kg dasatinib by oral gavage. Blood samples were obtained 2 h later and stained with the PY20 **(A, B)** or the 4G10 **(C, D)** anti-phosphotyrosine antibodies along with isotype controls. Where indicated, the anti-phosphotyrosine antibodies were presaturated with 4 mM soluble phosphotyrosine. Representative flow cytometric histograms **(A, C)** or geometric mean fluorescence intensity values normalized to those of vehicle-treated samples stained with anti-phosphotyrosine antibodies without any presaturation with their antigens **(B, D)** are shown. Bar graphs show mean and SEM from 5 **(B)** or 12 **(D)** mice per treatment from 3 **(B)** or 6 **(D)** independent experiments. pTyr, phosphotyrosine; Iso, isotype.

As shown in Figure 5A, dasatinib treatment caused a strong reduction of the fluorescence signal when neutrophil tyrosine phosphorylation was tested using the PY20 anti-phosphotyrosine antibodies (*p* = 6.3 × 10^−13^). Importantly, however, a remaining tyrosine phosphorylation signal could still be observed in samples from dasatinib-treated mice. Quantification of this response (Figure 5B) revealed that dasatinib caused a 94.8% inhibition of neutrophil tyrosine phosphorylation under such conditions. The remaining fluorescence signal could be further reduced by pre-saturation of the anti-phosphotyrosine antibodies with soluble phosphotyrosine amino acids, suggesting that the effect of dasatinib on tyrosine phosphorylation was indeed incomplete (Figure 5B).

We have also performed similar experiments using the 4G10 anti-phosphotyrosine antibodies. As shown in Figures 5C, D, dasatinib also strongly reduced the tyrosine phosphorylation signal of neutrophils detected by this approach (*p* = 9.0 × 10^−6^).

Taken together, our experiments revealed robust reduction of the intracellular tyrosine phosphorylation of neutrophils 2 h after oral administration of 50 mg/kg dasatinib, indicating that our approach is indeed able to rapidly detect the effect of orally administered tyrosine kinase inhibitors. These results at the same time provide an independent pharmacological validation of our fluorescence-based neutrophil tyrosine phosphorylation assay.

### Dose-response relationship of dasatinib treatment

Having been able to directly measure the effect of oral dasatinib treatment on intracellular tyrosine phosphorylation of circulating neutrophils, we next decided to test the dasatinib dose-response relationship during such experiments. To this end, dasatinib doses ranging from 1 to 50 mg/kg was administered to wild-type mice by oral gavage, followed by analysis of neutrophil tyrosine phosphorylation from a drop of blood taken 2 h later. The PY20 antibodies showed more robust and intensive fluorescence signal with better signal-to-noise ratio compared to the 4G10 clone (see Figures 2B, E) which prompted us to perform these experiments using only the PY20 anti-phosphotyrosine antibodies.

**FIGURE 6:**
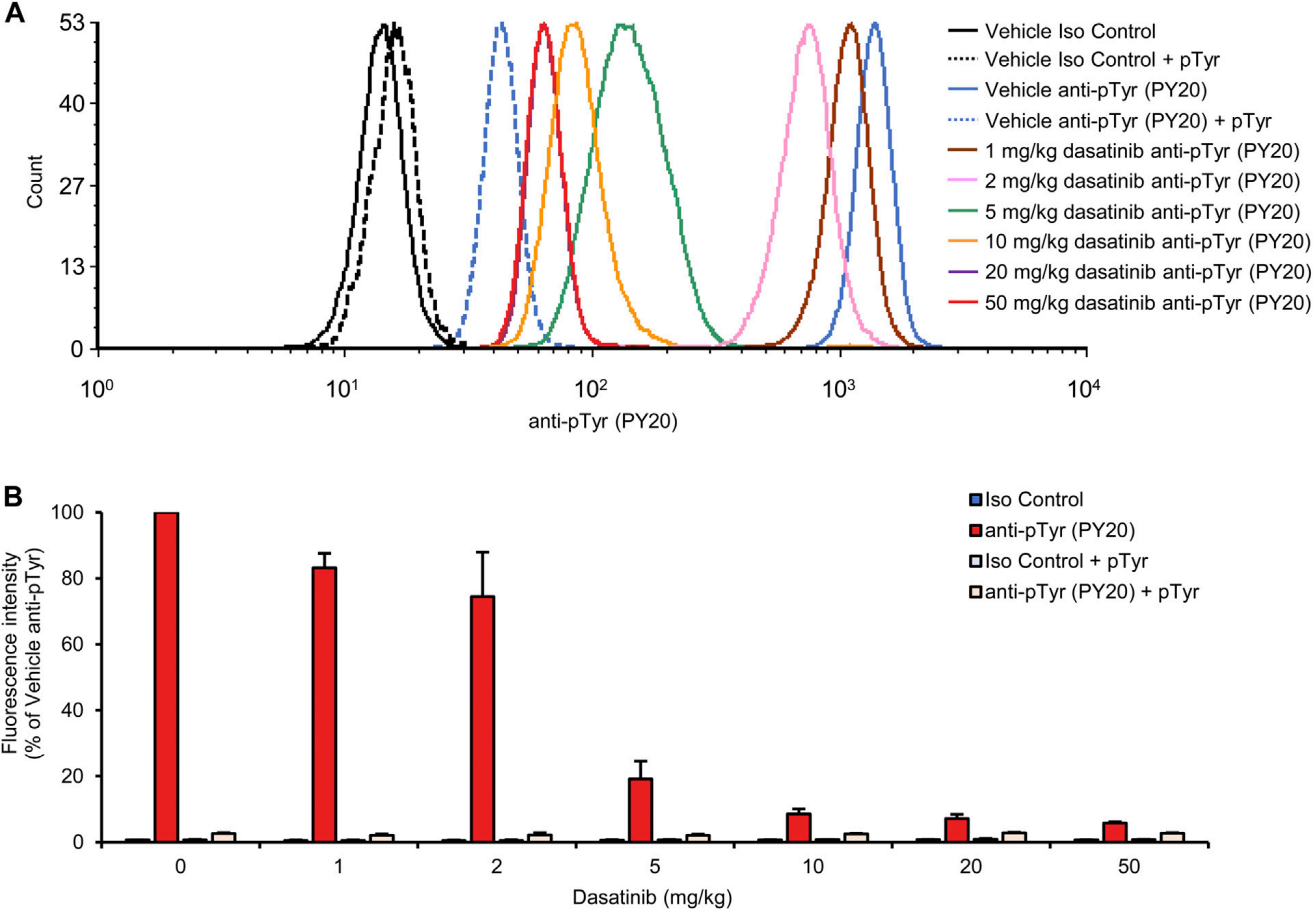
Dose-response relationship of dasatinib treatment. Wild-type mice were treated with vehicle or 1, 2, 5, 10, 20 or 50 mg/kg dasatinib by oral gavage. Blood samples were obtained 2 h later and stained with the PY20 anti-phosphotyrosine antibodies or isotype controls. Where indicated, the anti-phosphotyrosine antibodies were presaturated with 4 mM soluble phosphotyrosine. Representative flow cytometric histograms **(A)** or geometric mean fluorescence intensity values normalized to those of vehicle-treated samples stained with anti-phosphotyrosine antibodies without any presaturation with their antigens **(B)** are shown. The bar graph shows mean and SEM from 3-8 mice per treatment from at least three independent experiments. pTyr, phosphotyrosine; Iso, isotype.

As shown in the histogram in Figure 6A, increasing doses of dasatinib caused a gradual reduction of the fluorescence signal representing neutrophil tyrosine phosphorylation. There was a strong overlap between the curves corresponding to 20 and 50 mg/kg doses, suggesting that these doses caused similar levels of nearly complete inhibition. Quantification of the flow cytometry data (Figure 6B) revealed that half-maximal inhibition was achieved at a dasatinib dose of approx. 2.9 mg/kg, and a nearly complete (92%) inhibition was achieved at 10 mg/kg (*p* = 9.2 × 10^−12^; Dunnett’s *post hoc* test showed significant reduction at 5, 10, 20, and 50 mg/kg doses compared to anti-pTyr-stained vehicle-treated samples, *p* = 8.6 × 10^−6^). Again, the fluorescence signal observed in samples from dasatinib-treated mice could be further reduced by presaturation of the anti-phosphotyrosine antibodies with soluble phosphotyrosine amino acids, indicating that the remaining fluorescence signal is still caused by intracellular tyrosine phosphorylation of neutrophils.

### Kinetic analysis of the effect of dasatinib

One of the advantages of our assay is the detailed and quantitative analysis of neutrophil tyrosine phosphorylation from a single drop of peripheral blood, without sacrificing the mice. This allows us to follow tyrosine phosphorylation over a period of time, providing information about the kinetics of the biological effects of the inhibitors used. To exploit this opportunity, we have tested neutrophil tyrosine phosphorylation in mice treated with varying dasatinib doses at multiple time points over a 24-h period. Mice were treated with dasatinib doses ranging between 5 and 50 mg/kg and the intracellular tyrosine phosphorylation of neutrophils was tested immediately before, as well as 2, 5, 8 and 24 h after dasatinib treatment. Again, these experiments were only performed using the PY20 anti-phosphotyrosine antibodies. To facilitate the comparison of the data, fluorescence intensities were normalized to values of anti-phosphotyrosine antibody-treated samples of vehicle control mice at each time point.

**FIGURE 7:**
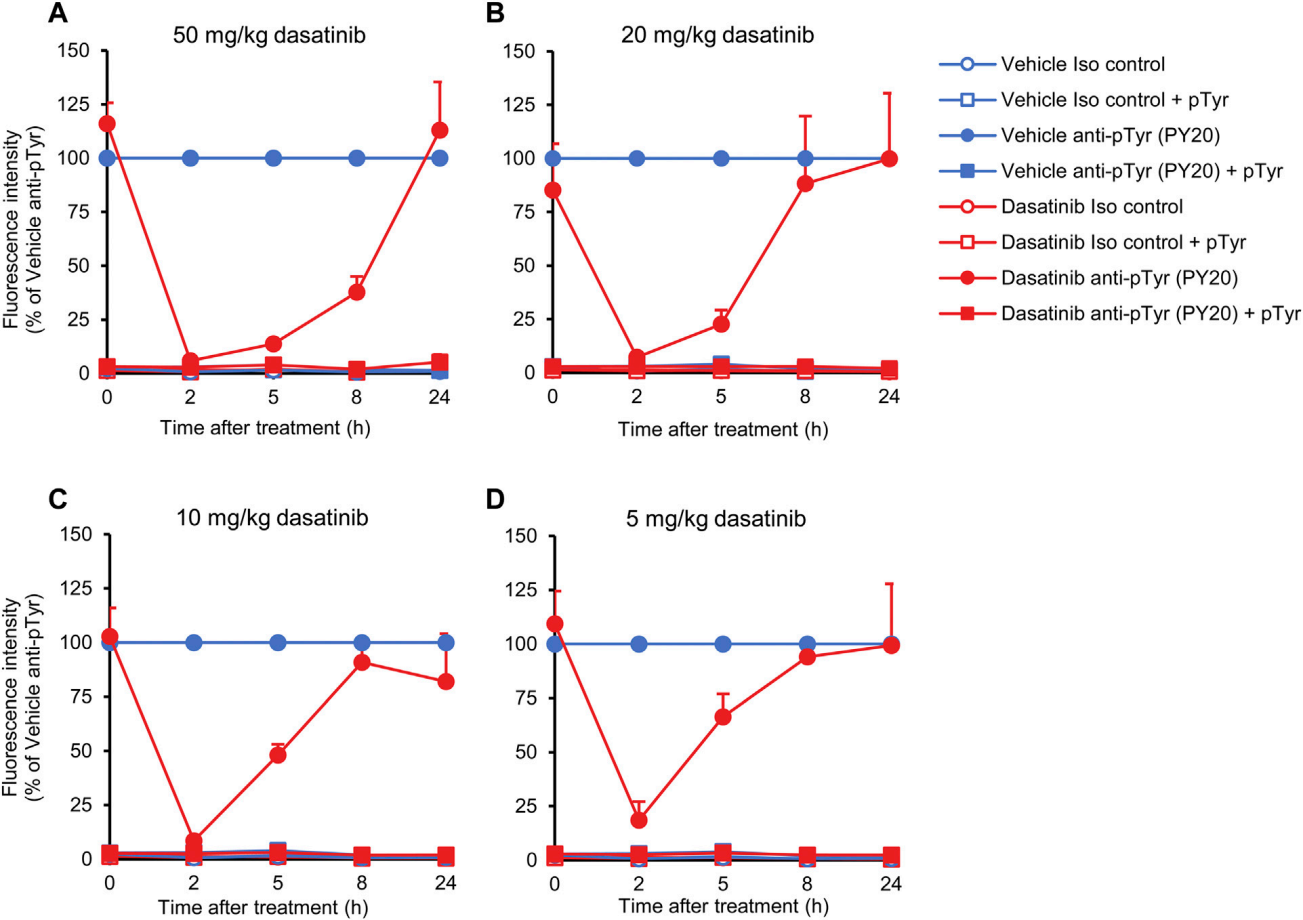
Kinetic analysis of the effect of dasatinib treatment. Wild-type mice were treated with vehicle or or 50 **(A)**, 20 **(B)**, 10 **(C)**, 5 mg/kg **(D)** dasatinib by oral gavage. Blood samples were obtained immediately before (0 time point) or 2, 5, 8 or 24 h later and stained with the PY20 anti-phosphotyrosine antibodies or isotype controls. Where indicated, the anti-phosphotyrosine antibodies were presaturated with 4 mM soluble phosphotyrosine. Geometric mean fluorescence intensity values obtained from the indicated mice and normalized to those of vehicle-treated samples stained with anti-phosphotyrosine antibodies without any presaturation with their antigens are shown. The graphs show mean and SEM from 3-8 mice per treatment from at least three independent experiments. SEM values of the normalized isotype control samples (with or without soluble phosphotyrosine pretreatment) were extremely low. pTyr, phosphotyrosine; Iso, isotype.

As shown in Figure 7A, after a nearly complete inhibition of neutrophil tyrosine phosphorylation by 50 mg/kg dasatinib at the 2-h time point (*p* = 1.3 × 10^−13^), the inhibitory effect of dasatinib remained very strong at the 5-h (*p* = 2.2 × 10^−3^) and still more than half-maximal at the 8-h time points (*p* = 8.4 × 10^−3^). However, neutrophil tyrosine phosphorylation returned to normal levels by the 24-h time point (*p* = 0.61). Decreasing the dose of dasatinib to 20 or 10 mg/kg hardly affected the inhibitory effect at the 2-h time point (*p* = 6.9 × 10^−10^ and *p* = 8.4 × 10^−10^, respectively), whereas the inhibitory effect was significant but less pronounced at the 5 mg/kg dose (Figures 7B–D; *p* = 4.3 × 10^−8^). This is in line with the saturation of the effect of dasatinib at the 2-h time point at or above 10 mg/kg dasatinib (see Figure 6B). However, a very different picture could be seen at later time points. At 5 h, a very clear difference between the effect of the different dasatinib doses was observed, indicating still near-maximal inhibition by the 50 mg/kg dose, but weaker inhibition at 10 and 20 mg/kg (*p* = 0.22 and *p* = 0.045), and hardly any inhibition at 5 mg/kg (*p* = 0.49). At 8 h, only the 50 mg/kg dose showed a substantial inhibitory effect, which was hardly stronger than 50%. Importantly, all fluorescence signals could be practically completely abrogated by pretreatment of the anti-phosphotyrosine antibodies with soluble phosphotyrosine amino acids, indicating the specificity of the signal for tyrosine phosphorylation.

Taken together, the above experiments indicate gradually decreasing effects of dasatinib after more than 2 h from oral administration, and at the same time demonstrate the suitability of our assay to follow the effect of oral tyrosine kinase inhibitors in a kinetic manner.

## Discussion

Tyrosine kinases are major targets of the development of novel pharmaceuticals for the treatment of a number of various diseases. Their main advantages as therapeutic targets include their critical role in major human diseases, their enzymatic activity allowing easy targeting by small-molecule inhibitors, their diversity providing opportunity for targeting specific biological processes, as well as the very substantial experience in developing small-molecule tyrosine kinase inhibitors. The main medical fields where such inhibitors can be used include the various types of cancer, as well as autoimmune and inflammatory diseases.

An important step of the development of small-molecule tyrosine kinase inhibitors is the *in vitro* analysis of their effects on various cellular functions using cell lines or primary cells. However, those experiments are often unable to predict the *in vivo* effects of the inhibitors, their permeability across the various biological barriers (e.g., intestinal absorption, transport across the plasma membrane of target cells), their removal from the circulation by degradation or excretion, and their overall *in vivo* efficacy. Therefore, these characteristics need to be tested using other approaches such as administration of drug candidates to experimental animals. However, measuring drug concentrations in the circulation may be rather difficult and require very unique experience/equipment because of the difficulties of testing specific organic compounds in a complex biological environment. The efficacy of the inhibitors also needs complex and time consuming *in vivo* disease models. Those *in vivo* models often rely on the use of a large number of experimental animals and may require large inhibitor quantities which necessitate scaling up the chemical synthesis using entirely new synthesis pathways. Alternatively, some of the *in vivo* characteristics of the inhibitors (e.g., their pharmacokinetic properties) can be approximated by *in vitro* assays. However, those assays have their own substantial limitations and should also be followed by conventional *in vivo* assays. Taken together, the transition of the development of tyrosine kinase inhibitors to *in vivo* analysis is rather difficult and consumes substantial time and resources.

Our assay system provides substantial advantages over the above scenario. Our experiments test the basal *in vivo* tyrosine phosphorylation of neutrophils as an approximation of *in vivo* tyrosine phosphorylation in cells of hematopoietic origin. This assay is suitable for the rapid detection and precise quantification of the biochemical effect of orally administered tyrosine kinase inhibitors. It therefore provides an approximation of the transportation of the inhibitors across biological membranes and barriers, it is intrinsically corrected for factors (e.g., metabolism, excretion) that affect the biological availability of the given compounds, and it allows an approximate assessment of the biological effect of the inhibitors, at least at the biochemical level. In addition, our approach requires very low inhibitor quantities (sufficient for a single oral dose), therefore it does not require synthesis of large inhibitor quantities, giving the opportunity to test the *in vivo* effect of a larger number of inhibitors in early preclinical phases. Our assay procedure is also relatively simple and is based on widely available reagents and equipment, therefore no complex assay development is needed. The fact that quantitative results can be obtained in not more than a few hours after oral inhibitor administration may also provide substantial benefit. Finally, the fact that the animals do not need to be sacrificed for the assay procedure allows more efficient use of experimental animals (e.g., during kinetic assays) and a better implementation of the principle of the 3R (replacement, reduction and refinement) of the humane use of experimental animals. Taken together, we believe that the experimental approach described here provides a number of advantages over the conventional steps of the development of tyrosine kinase inhibitor drug candidates and may therefore be a potentially viable addition to the methodological arsenal of the transition from the *in vitro* to the *in vivo* stages of drug development, allowing a more time- and cost-efficient approach with a shorter time-to-clinic interval.

A number of our experiments served for the validation of our approach at different conceptual levels. The use of two different anti-phosphotyrosine antibodies and a suitable isotype-matched control (Figure 2) provided strong evidence that our approach does indeed detect tyrosine phosphorylation of circulating neutrophils. This was further confirmed by analyzing the competitive effect of presaturing the anti-phosphotyrosine antibodies with their cognate antigens, either in the form of soluble phosphotyrosine amino acids or as short tyrosine-phosphorylated oligopeptides (Figure 3). The analysis of *Hck* ^−/−^ *Fgr* ^−/−^ *Lyn* ^−/−^ mice (Figure 4) provided a genetic confirmation for the specificity of our assay for intracellular tyrosine phosphorylation. Finally, the effect of dasatinib (Figure 5) indicated, using a true pharmacological approach, that our assay is indeed capable of testing the effects of orally administered tyrosine kinase inhibitors. Taken together, a number of independent approaches confirmed the suitability of our assay for testing *in vivo* tyrosine phosphorylation of neutrophils.

Our experiments also revealed the capability of our approach for detailed analysis of the dose-response relationship and kinetics of orally administered tyrosine kinase inhibitors. Indeed, experiments shown in Figures 6, 7 indicate that very detailed information on those aspects can be efficiently obtained using our assay system.

A clear limitation of our assay is that it focuses on general tyrosine phosphorylation within neutrophils, therefore it is only suitable for the analysis of inhibitors with an intended effect on those cells or at least the myeloid compartment. Nevertheless, given the major challenges of treating hematological malignancies and inflammatory diseases, testing tyrosine phosphorylation in neutrophils may be a suitable strategy for the development of a number of drug candidates.

Our experiments may also open up avenues of development of further assays based on similar principles. Such improvements may proceed along various different directions. Using antibodies labeling populations other than neutrophils, it would be relatively easy to extend our assay to the analysis of other circulating leukocytes such as B- and T-cells, monocytes, eosinophils or basophils. We should also be able to test intracellular tyrosine phosphorylation of various leukocyte subpopulations, including different subpopulations of B-cells, T-cells and monocytes. With appropriate selection of fluorochromes and multi-channel flow cytometry, such details could also be obtained from the same single samples labeled by a number of different antibodies, optimizing the use of experimental animals. Another possible direction of further assay development would be the use of phosphorylation-specific antibodies of given intracellular substrates. That way, we should be able to test the effect of inhibitors on specific tyrosine phosphorylation pathways rather than on the general tyrosine phosphorylation response. Although such improvements of our assay would be relatively straightforward, they would also open up a number of new methodological and conceptual issues that we felt are beyond the scope of the experimental work described in this paper.

Taken together, we believe that our experimental approach of flow cytometric analysis of basal tyrosine phosphorylation of neutrophils may provide a useful addition to the current tools of the transition of the development of tyrosine kinase inhibitors from an *in vitro* to an *in vivo* developmental phase. Our assay also has the potential to inspire the development of further conceptually similar assays that may extend well beyond the analysis of drugs targeting overall tyrosine phosphorylation in the myeloid compartment.

## Data Availability

The primary data supporting the conclusions of this study are available from the corresponding author upon reasonable request.
